# Auditory and Tinnitus Outcomes of Vibrant Soundbridge Implantation with the Incus Short Process Coupler in Older Male Veterans

**DOI:** 10.3390/brainsci16040423

**Published:** 2026-04-17

**Authors:** Chul Ho Jang, Do Yeon Kim

**Affiliations:** 1 Department of Otolaryngology, Gwangju Veterans Hospital, Gwangju 62284, Republic of Korea; ehdus0905@bohun.or.kr; 2 Department of Otolaryngology, Chonnam National University Medical School, Gwangju 61469, Republic of Korea

**Keywords:** Vibrant Soundbridge, active middle ear implant, short process coupler, sensorineural hearing loss, tinnitus, auditory rehabilitation, older male veterans

## Abstract

**Highlights:**

**What are the main findings?**

- VSB implantation with the incus short process (SP) coupler provides significant high-frequency hearing gain in older adults with bilateral sloping sensorineural hearing loss.
- The procedure resulted in a mean functional hearing gain of 21.8 dB, with the greatest improvement at speech-critical frequencies (2–4 kHz).

**What are the implications of the main findings?**

- Patient-reported hearing quality improved significantly across all Speech, Spatial, and Qualities of Hearing Scale (SSQ) domains.
- Tinnitus Handicap Inventory scores decreased by 60%, indicating substantial tinnitus relief following implantation.
- The short process coupler technique enables efficient surgery with minimal complications, making it suitable for elderly patients.

**Abstract:**

**Background**: Active middle ear implants (AMEIs) provide an alternative auditory rehabilitation strategy for patients who cannot tolerate conventional hearing aids. However, clinical data regarding the outcomes of Vibrant Soundbridge (VSB) implantation using the incus short process (SP) coupler in older adults remain limited. **Objective:** This study aimed to evaluate the audiological outcomes, patient-reported hearing benefits, tinnitus improvement, and surgical safety of VSB implantation using the SP coupler in older adults with bilateral sloping sensorineural hearing loss. **Methods**: This retrospective study included 45 older male veterans (mean age 76.1 ± 5.3 years) with bilateral sloping sensorineural hearing loss who underwent unilateral VSB implantation with the SP coupler between 2019 and 2023. Functional hearing gain was assessed using preoperative and postoperative sound-field pure-tone thresholds. Patient-reported outcomes were evaluated using the Speech, Spatial and Qualities of Hearing Scale (SSQ) and the Tinnitus Handicap Inventory (THI). Operative characteristics and postoperative complications were also analyzed. **Results**: Mean operative time was 40.2 ± 8.7 min. Functional hearing gain increased progressively across speech-critical frequencies, reaching +20 dB at 2 kHz and +30 dB at 4 kHz. The mean four-frequency pure tone average improved from 57.4 ± 8.3 dB HL preoperatively to 35.6 ± 6.9 dB HL postoperatively (*p* < 0.001). All SSQ subdomains showed significant improvement (*p* < 0.001). THI scores decreased significantly from 43.2 ± 8.4 to 17.1 ± 6.2 (*p* < 0.0001), with clinically meaningful tinnitus improvement observed in 75.6% of patients. No major surgical complications occurred. **Conclusions**: Vibrant Soundbridge implantation using the incus short process coupler provides effective auditory rehabilitation for older adults with sloping sensorineural hearing loss. The procedure yields meaningful high-frequency hearing gain, improved hearing-related quality of life, and significant tinnitus reduction while maintaining a favorable surgical safety profile. Restoration of auditory input through active middle ear implantation may also contribute to improved central auditory processing in older adults.

## 1. Introduction

Sensorineural hearing loss (SNHL) is among the most prevalent sensory disabilities worldwide, with a disproportionate impact on older adults. Sloping high-frequency SNHL, the most common audiometric pattern in the geriatric population, particularly compromises speech intelligibility in noise and adversely affects quality of life, communication, and cognitive function [1,2]. Although conventional acoustic hearing aids remain the first-line intervention, a subset of older adults cannot tolerate them because of chronic otitis externa, ear canal irritation, occlusion effects, feedback, or inadequate high-frequency amplification. In some elderly patients receiving long-term anticoagulation therapy, recurrent ear canal irritation or bleeding associated with hearing-aid use may further limit tolerance [3]. Active middle ear implants (AMEIs) offer an effective alternative in such patients’ feedback, occlusion effects, or insufficient amplification in the high-frequency range where the hearing loss is most severe [4,5]. Active middle ear implants (AMEIs) offer an effective alternative in such patients by delivering mechanical vibrations directly to the ossicular chain, bypassing the outer ear and thereby eliminating feedback and the occlusion effect while providing superior high-frequency amplification [4]. The Vibrant Soundbridge (VSB; MED-EL, Innsbruck, Austria) is the only currently available AMEI and has been in clinical use since 1996. It consists of a partially implantable system: an internally implanted vibrating ossicular prosthesis (VORP) and an externally worn audio processor (SAMBA 2). At its core is the floating mass transducer (FMT), a miniaturized electromagnetic transducer that converts audio signals received via transcutaneous inductive coupling from the audio processor into mechanical vibrations, which are then directly transmitted to the middle ear ossicles [1,6]. The FMT can be coupled to various ossicular structures depending on middle ear anatomy and hearing loss type, including the long process (LP) of the incus, the round window, the stapes head, or the short process (SP) of the incus [4]. For patients with SNHL, the incus vibroplasty approach, in which the FMT is attached to the incus, is the standard coupling modality [5,7,8].

The conventional LP vibroplasty requires a wide posterior tympanotomy approach to provide adequate visualization and access for FMT clip attachment to the long process of the incus. This approach, while effective, is associated with a risk of injury to the facial nerve or chorda tympani, a longer operative time, and greater surgical complexity [6]. The introduction of the SP coupler with the third-generation VORP 503 implant (available since 2015) represented a significant technical advancement, enabling attachment of the FMT to the body and short process of the incus via an endaural or retroauricular approach without the need for posterior tympanotomy. Studies have demonstrated that SP vibroplasty reduces operative time by approximately 35%, with comparable audiological outcomes to LP vibroplasty [6,9,10]. The SP coupler thus offers a simpler, faster, and less morbid surgical technique, which is particularly relevant in older patients with medical comorbidities. Despite its clinical relevance, evidence specifically addressing VSB outcomes with the SP coupler in older adult populations with bilateral sloping SNHL remains limited. The majority of candidates for VSB implantation are older than 55 years [3], yet outcome studies in this population often group patients by hearing loss type rather than age, potentially obscuring age-specific patterns of benefit, tolerance, and complication. In addition, tinnitus is a prevalent comorbidity in older adults with SNHL; however, the effect of VSB implantation on tinnitus in this population has received limited systematic attention [8,11]. Furthermore, the growing use of anticoagulant therapy in the elderly cardiac population raises practical perioperative concerns, and the interaction between anticoagulant use and middle ear implant surgery outcomes has not been well characterized.

To our knowledge, this study represents one of the largest single-center analyses focusing specifically on VSB implantation using incus short process coupler in an elderly veteran population, with particular attention to tinnitus outcomes and perioperative considerations such as anticoagulant therapy.

The purpose of this study is to evaluate the surgical, audiological, and patient-reported outcomes of VSB MEI with the SP coupler in older adults with bilateral sloping SNHL, with particular focus on functional hearing gain, speech-spatial quality of hearing, tinnitus relief, and surgical safety.

## 2. Patients and Methods

### 2.1. Study Design and Ethics

This retrospective observational study was approved by the Institutional Review Board (IRB) of Gwangju Veterans Hospital (IRB No. GVH-IRB-2024-15-2) and conducted in accordance with the principles of the Declaration of Helsinki. Waiver of informed consent was granted by the IRB given the retrospective nature of the electronic chart analysis.

### 2.2. Study Population

Between January 2019 and December 2023, 45 consecutive male patients (mean age 76.12 ± 5.3 years; range 65–85 years) with bilateral sloping SNHL who underwent unilateral VSB MEI implantation with the incus SP coupler at Gwangju Veterans Hospital were enrolled. Inclusion criteria were: (1) bilateral sloping SNHL with air-conduction pure tone average (PTA) of 40–70 dB HL at 1–4 kHz in the implanted ear; (2) bone conduction thresholds within the VSB SNHL indication range; (3) failure to benefit adequately from conventional hearing aids (HA trial ≥ 3 months); (4) age ≥ 65 years; and (5) open-set speech understanding ≥ 50% at most comfortable listening level. Exclusion criteria included conductive or mixed hearing loss, active middle or outer ear disease, prior ear surgery (excluding myringotomy), and inability to comply with follow-up. Eight patients (17.8%) were receiving long-term oral anticoagulant therapy (warfarin, *n* = 5; direct oral anticoagulants, *n* = 3) for atrial fibrillation or deep vein thrombosis.

### 2.3. Device Description

The VIBRANT SOUNDBRIDGE VORP 503 (MED-EL, Innsbruck, Austria) was used in all cases. The implanted component consists of a demodulator housing containing the receiver coil and electronics, a conductor link, and the FMT. The SP coupler (Incus-SP-Coupler) attaches the FMT to the body and short process of the incus using a standardized single-point crimping mechanism, securing the FMT without requiring posterior tympanotomy. The external audio processor (SAMBA 2) communicates wirelessly with the implant via transcutaneous inductive coupling. MRI compatibility: the VORP 503 is MR Conditional at 1.5 Tesla; however, fibrosis forming around the FMT over time may limit diagnostic quality of postoperative MRI scans of the temporal region [10].

### 2.4. Surgical Technique

**Figure 1:**
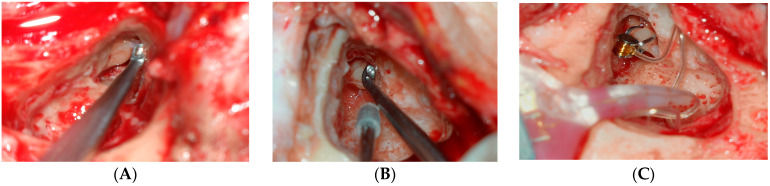
Intraoperative photographs of VSB implantation with the short process (SP) coupler. (**A**) Removal of bony overhang adjacent to the incus short process using a microcurett to facilitate floating mass transducer (FMT) access. (**B**) Silicone-tip suction device used during FMT positioning to minimize mucosal trauma. (**C**) FMT secured to the short process of the incus via the SP coupler. The conductor link is visible leading to the implanted VORP 503 housing.

All procedures were performed under general anesthesia by a single experienced otolaryngologist (C.H.J.) A standard retroauricular incision was first performed, followed by cortical mastoidectomy. A sufficiently wide epitympanotomy was then created to achieve complete visualization of the incus body and its short process. A microcurett was systematically employed to carefully remove any bony overhang adjacent to the incus body and short process to ensure adequate visualization and unimpeded FMT placement (Figure 1A). A silicone-tip suction device was used for gentle aspiration in proximity to the ossicular chain to prevent mucosal trauma during FMT positioning (Figure 1B). For coupling, a dedicated short process coupler (e.g., SP2 coupler) is utilized to secure the Floating Mass Transducer (FMT). The FMT should be aligned such that its vibratory axis is oriented perpendicular to the stapes footplate to optimize mechanical energy transmission. For coupling, a dedicated short process coupler (e.g., SP2 coupler) was utilized to secure the Floating Mass Transducer (FMT). The FMT should be aligned such that its vibratory axis is oriented perpendicular to the stapes footplate to optimize mechanical energy transmission. The VORP 503 was implanted in the subperiosteal pocket posterior to the auricle. The conductor link was routed and secured with titanium screws to prevent tension or displacement. The FMT was then clamped securely onto the short process of the incus using the SP coupler (Figure 1C). The wound was closed in layers. For anticoagulated patients, anticoagulant therapy was managed perioperatively according to institutional hematology protocol: warfarin was bridged with low-molecular-weight heparin, and direct oral anticoagulants were held for 24–48 h preoperatively and resumed 24 h postoperatively. The device was typically activated 6 to 8 weeks post-op once the surgical site has fully healed and swelling has subsided.

### 2.5. Outcome Measures

The primary outcome was functional hearing gain, defined as the difference between preoperative unaided and postoperative aided sound-field PTA at 0.25, 0.5, 1, 2, 4, and 8 kHz. Sound-field audiometry was performed using calibrated warble tones delivered via calibrated loudspeakers. Testing was performed with VSB activated in the implanted ear only, while the contralateral ear was occluded and masked with narrowband noise when necessary to minimize cross-hearing effects. No contralateral hearing aid was used during the test.

Secondary outcomes were: (1) The Speech, Spatial, and Qualities of Hearing Scale (SSQ), a validated 12-item patient-reported outcome measure (PROM) scored from 0 (maximum disability) to 10 (no disability), covering speech hearing, spatial hearing, and qualities of hearing subscales [12]. (2) All enrolled patients reported chronic subjective tinnitus associated with sensorineural hearing loss prior to implantation, and tinnitus severity was assessed using the Tinnitus Handicap Inventory (THI) both before and after surgery. THI, a validated 25-item questionnaire scored 0–100, with higher scores indicating greater tinnitus handicap [13]. (3) Operative time and surgical complications. (4) Device-related adverse events and follow-up observations.

### 2.6. Statistical Analysis

Statistical analyses were performed using GraphPad Prism 8.0. Preoperative and postoperative audiometric, SSQ, and THI scores were compared using the paired-samples *t*-test. Data are presented as mean ± standard deviation (SD). A *p*-value < 0.05 was considered statistically significant.

## 3. Results

### 3.1. Patient Demographics and Surgical Characteristics

The demographic and surgical characteristics of the 45 patients are summarized in Table 1. All patients were male, consistent with the predominantly male veteran population of this institution. Mean age at implantation was 76.12 ± 5.3 years (range 65–85 years). The most common indication was bilateral sloping SNHL with inadequate benefit from conventional hearing aids (*n* = 38, 84.4%), followed by hearing aid intolerance due to chronic otitis externa or ear canal discomfort (*n* = 7, 15.6%). Mean follow-up duration was 10.8 ± 3.6 months (range 7–12 months).

Mean operative time was 40.2 ± 8.7 min. Intraoperative use of the microcurett for bony remodeling near the incus short process was required in 31 patients (68.9%) to ensure unimpeded FMT placement. The silicone-tip suction device was used routinely in all cases. The SP coupler was successfully attached to the incus short process in all patients on the first attempt, without the need for posterior tympanotomy in any case.

### 3.2. Audiological Outcomes

**Figure 2:**
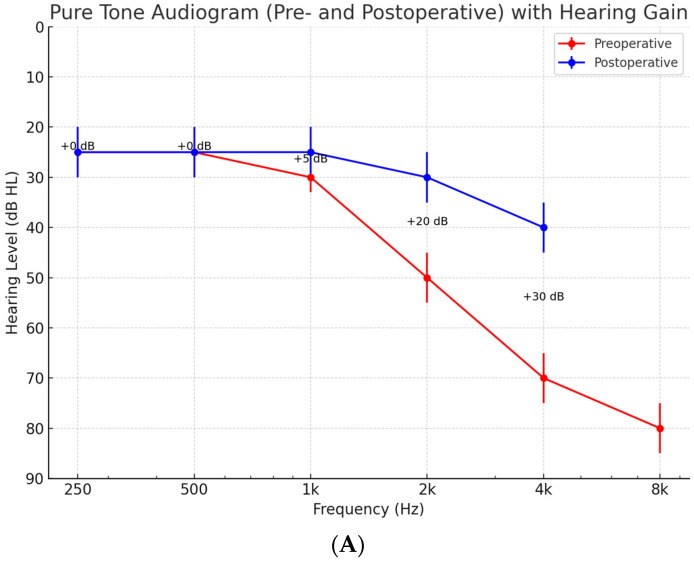

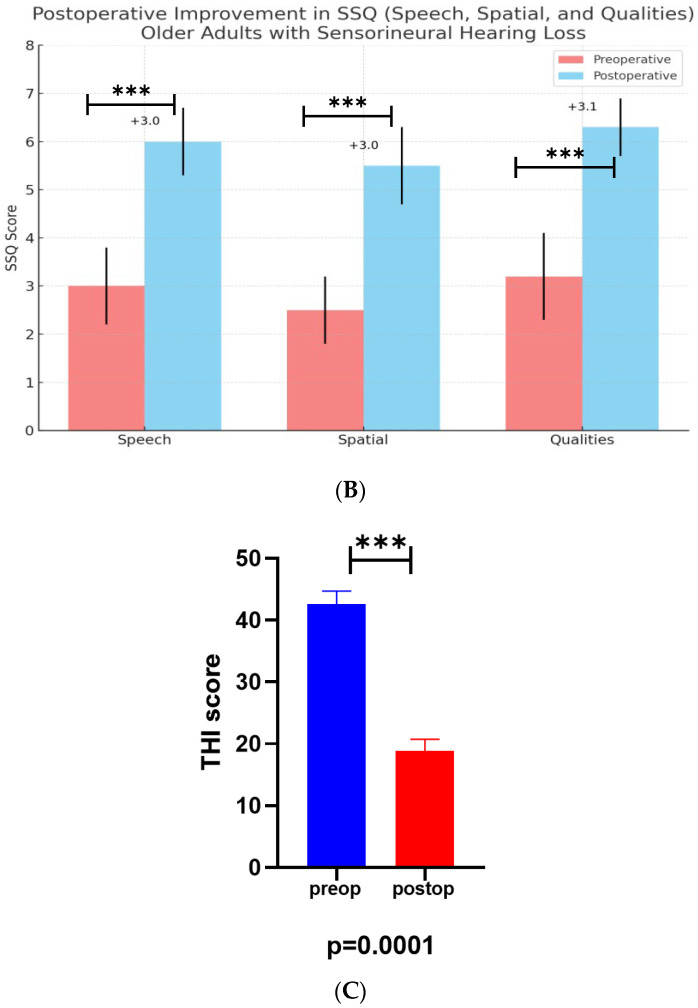
(**A**) Sound-field pure-tone thresholds before and after Vibrant Soundbridge implantation. Preoperative unaided thresholds are shown in red and postoperative VSB-aided thresholds in blue. Mean functional hearing gain at each frequency is indicated. Error bars represent ±1 SD. (**B**) Improvement in Speech, Spatial, and Qualities of Hearing Scale (SSQ) subscores after VSB implantation. Preoperative scores are shown in red and postoperative scores in blue. Error bars represent ±1 SD. *** *p* < 0.001 (paired *t*-test). (**C**) **Tinnitus Handicap Inventory (THI) scores before and after VSB implantation.** Preoperative scores are shown in red and postoperative scores in blue. Error bars represent ± 1 SD. *** *p* = 0.0001 (paired *t*-test).

Table 2 and Figure 2A present the preoperative unaided and postoperative aided sound-field thresholds. In the low-frequency range (250–500 Hz), no functional hearing gain was observed (mean gain 0 dB), consistent with the flat low-frequency thresholds characteristic of sloping SNHL. Significant functional gain was demonstrated at speech-critical frequencies: +5.0 ± 3.8 dB at 1 kHz (*p* = 0.021), +20.0 ± 5.4 dB at 2 kHz (*p* < 0.001), and +30.2 ± 6.1 dB at 4 kHz (*p* < 0.001). The mean 4-frequency PTA (0.5, 1, 2, 4 kHz) improved from 57.4 ± 8.3 dB HL unaided to 35.6 ± 6.9 dB HL aided (mean functional gain 21.8 ± 5.2 dB; *p* < 0.001). Bone conduction thresholds remained stable at all follow-up visits, with a mean shift in less than 5 dB at any single frequency, indicating no significant cochlear damage attributable to the implanted device.

### 3.3. Speech, Spatial, and Qualities of Hearing (SSQ)

All three SSQ subscales demonstrated statistically significant improvement following VSB activation (Figure 2B). The Speech subscale score improved from 3.0 ± 0.9 preoperatively to 6.0 ± 0.8 postoperatively (mean improvement +3.0; *p* < 0.001). The Spatial subscale improved from 2.5 ± 0.8 to 5.5 ± 0.9 (mean improvement +3.0; *p* < 0.001). The Qualities subscale improved from 3.1 ± 1.0 to 6.2 ± 0.9 (mean improvement +3.1; *p* < 0.001). These improvements indicate that patients reported substantially less disability across all domains of hearing function after MEI activation.

### 3.4. Tinnitus Outcomes

The mean preoperative THI score was 43.2 ± 8.4 (indicating moderate tinnitus handicap), which decreased significantly to 17.1 ± 6.2 postoperatively (indicating mild tinnitus handicap; mean improvement 26.1 ± 7.3; *p* = 0.0001; Figure 2C). A clinically meaningful THI reduction (≥20 points) was observed in 34 patients (75.6%). No patient reported worsening of tinnitus after VSB implantation.

### 3.5. Complications and Adverse Events

No major intraoperative or postoperative complications occurred in any patient. Device dislodgement, facial nerve injury, perilymphatic fistula, or wound infection were not observed. Among the 8 patients on anticoagulant therapy, all developed transient postoperative hemotympanum characterized by middle ear fullness and a conductive component on audiometry. These cases resolved spontaneously within 4–8 weeks without surgical intervention. None of the anticoagulated patients required re-exploration or drainage. All patients resumed anticoagulant therapy within 24 h postoperatively.

An important device-related observation was encountered in 3 patients (6.7%) who required postoperative temporal bone or brain MRI during the follow-up period. In all three cases, fibrosis surrounding the FMT resulted in significant image artifact extending beyond the middle ear, substantially limiting the diagnostic quality of temporal bone and adjacent brain MRI sequences. These findings were managed with radiologist consultation and modified MRI protocols, but diagnostic limitations persisted, consistent with prior reports regarding FMT-related MRI artifact [14].

## 4. Discussion

This retrospective study reports the outcomes of VSB MEI with the SP coupler in 45 older adult male patients with bilateral sloping SNHL treated at a single veterans hospital. Our findings demonstrate that the SP coupler approach achieves meaningful audiological benefit in the speech-critical frequency range, significant improvements in speech-spatial quality of hearing across all SSQ domains, and substantial tinnitus reduction, with a short operative time and a favorable safety profile.

### 4.1. Audiological Outcomes and the SP Coupler

The audiological profile observed in this study is characteristic of VSB SP vibroplasty for sloping SNHL. The absence of functional gain at 250–500 Hz reflects the well-established frequency-dependent attenuation inherent to the FMT-incus coupling mechanism and the audiometric configuration of sloping SNHL, in which low-frequency thresholds are near-normal and do not require amplification. The progressive functional gains at 1 kHz (+5 dB), 2 kHz (+20 dB), and 4 kHz (+30 dB) correspond precisely to the frequencies of greatest hearing loss, demonstrating that the FMT efficiently delivers targeted amplification to the impaired high-frequency cochlear region. This is clinically significant because it directly addresses the sloping pattern of loss, which creates the greatest difficulty with consonant perception and speech understanding in noise.

These results are consistent with published data on VSB SP vibroplasty. Edlinger et al. [4] reported a mean functional gain of 17.19 ± 5.75 dB in patients receiving the SP coupler, which was not significantly different from LP coupler recipients (20.91 ± 9.77 dB; *p* > 0.05), confirming audiological equivalence between the two coupling modalities. Schraven et al. [9] similarly demonstrated comparable audiological outcomes between SP and LP vibroplasty in a retrospective analysis of 36 patients, while the SP approach was associated with a 35% reduction in operative time and shorter hospital stay. Rahne et al. [10] in a European multicenter analysis of third-generation vibroplasty couplers found no significant difference in audiological performance among LP, SP, and round window couplers, supporting the principle that the choice of coupler should be guided primarily by individual anatomy and surgical accessibility rather than expected audiological outcome.

### 4.2. Surgical Technique: Value of Microcurett and Silicone Suction

Two intraoperative techniques merit specific comment as they were integral to the success of SP coupler implantation in our cohort. First, bony remodeling adjacent to the incus short process using a microcurett was required in 68.9% of cases. In older adults, bony sclerosis or overhang of the posterior osseous ear canal wall can obstruct direct access to the incus body and short process, particularly when posterior tympanotomy is deliberately avoided. The microcurett provides precise, controlled bone removal under microscopic visualization without the thermal risk and acoustic trauma associated with drilling, thereby protecting the ossicles and the cochlea. This technique has been described as an important adjunct in SP coupler implantation, especially in anatomically challenging cases [9].

Second, the routine use of a silicone-tip suction device during FMT positioning effectively prevented mucosal laceration and middle ear bleeding. In older patients who may be taking anticoagulants or who have more friable middle ear mucosa due to chronic hearing loss and limited tissue regeneration capacity, gentle surgical technique is particularly important. The silicone-tipped design prevents inadvertent aspiration of the tympanic membrane or ossicular ligaments and allows safe retraction during FMT clip attachment.

### 4.3. Speech-Spatial Quality of Hearing and Quality of Life

The improvement in all three SSQ subscales (Speech +3.0, Spatial +3.0, Qualities +3.1) reflects broad-based functional hearing benefit that extends beyond audiometric thresholds to encompass the real-world listening demands of daily life. The SSQ Speech subscale evaluates hearing in diverse background noise conditions and multi-talker settings; the Spatial subscale assesses directional hearing and sound source localization; and the Qualities subscale reflects signal clarity, naturalness, and segregation ability. Improvements in all three dimensions indicate that VSB with the SP coupler benefits patients not only in terms of audibility but also in terms of the complex perceptual processes that determine social and communicative function.

These findings are consistent with data from active MEI outcome studies using the SSQ. Salamah et al. [15] reported significant improvements in all SSQ12 domains following VSB implantation in patients with mixed hearing loss, and Lorente-Piera et al. [16] confirmed that subjective hearing benefit measured by SSQ consistently correlated with audiometric functional gain in a large single-center cohort. For older adults, improvements in the Spatial subscale are especially relevant because degraded directional hearing impairs the ability to monitor the acoustic environment, increasing the risk of fall-related accidents and social withdrawal.

### 4.4. Tinnitus and VSB Implantation

The significant reduction in THI scores (from 43.2 to 17.1; *p* = 0.0001) is an important finding in this cohort. Tinnitus is highly prevalent in older adults with SNHL, reported in up to 60–80% of patients with high-frequency loss [13]. In our cohort, nearly all patients reported tinnitus as a significant symptom at presentation, consistent with the relationship between SNHL and central auditory deafferentation leading to tinnitus generation. The mechanisms underlying tinnitus suppression with MEI are not fully established; however, the most widely accepted hypothesis is that restoration of adequate auditory input at the impaired frequencies through direct ossicular stimulation reduces central auditory gain and cortical reorganization associated with tinnitus perception [11].

These results align with those of Lee et al. [11], who reported significant reductions in THI scores and visual analogue scale (VAS) measures of tinnitus loudness, awareness, and annoyance in 16 adults with SNHL-associated tinnitus following VSB implantation with 12-month follow-up. A clinically meaningful THI reduction (≥20 points) was achieved in 75.6% of our patients, a rate comparable to the 70–80% reported in prior VSB tinnitus studies. These findings support the recommendation that MEI should be actively considered in older adults with sloping SNHL and comorbid tinnitus.

### 4.5. Anticoagulant Use and Hemotympanum

The occurrence of postoperative hemotympanum in all 8 anticoagulated patients (100% of anticoagulated cases) is clinically noteworthy and represents a previously under-reported complication in the MEI literature. Hemotympanum presents as middle ear fullness, a conductive component on postoperative audiometry, and otoscopic evidence of blood behind the tympanic membrane. In our cohort, all cases resolved spontaneously within 4–8 weeks without surgical drainage, suggesting that conservative management is appropriate in the absence of infection or severe conductive hearing deterioration. Anticoagulant therapy is common in the elderly population due to the high prevalence of atrial fibrillation, venous thromboembolism, and cardiovascular disease [17]. Surgeons planning VSB implantation in anticoagulated elderly patients should be aware of this complication, counsel patients preoperatively, and establish a standardized perioperative anticoagulation management protocol in collaboration with cardiology or hematology.

### 4.6. MRI Compatibility and FMT Fibrosis

The VORP 503 implant is MR Conditional at 1.5 Tesla. When MRI is required in patients with VSB implants, several strategies may help reduce artifact-related diagnostic limitations. These include the use of artifact-reduction MRI sequences, adjustment of slice orientation, and alternative imaging modalities such as CT when appropriate. Preoperative counseling regarding potential MRI limitations is also recommended [18]. However, a clinically important limitation observed in our cohort was the development of fibrosis around the FMT in 3 patients who required temporal bone or brain MRI during follow-up. This fibrosis, which forms as part of the foreign body response to the implanted FMT, generates significant image artifact that extends beyond the immediate vicinity of the device and may obscure adjacent structures on brain and temporal bone MRI [10]. Ernst et al. [19] have previously reported MRI complications with the VSB, noting that in some patients, scanner-related impulse noise and changes in FMT transfer function occur during scanning, with 2 of 63 implantees requiring transtympanal FMT repositioning after MRI. In our series, no FMT displacement occurred, but diagnostic quality was significantly impaired. Surgeons should preoperatively counsel VSB candidates who have conditions requiring periodic neurological or brain imaging about this limitation, and interdisciplinary communication with the radiologist should be established prior to any postoperative MRI.

### 4.7. Limitations

This study has several limitations. The retrospective single-center design limits generalizability, and the absence of a control group (e.g., conventional hearing aid users or LP coupler recipients) precludes direct comparative conclusions. The exclusively male cohort, representative of this veterans institution, limits applicability to the broader elderly SNHL population including women. The relatively short mean follow-up of 10.8 months does not capture long-term outcomes, including the potential for progressive hearing deterioration requiring cochlear implantation [20]. Objective assessments of sound localization and speech perception in noise were not performed in this retrospective study. Future studies should incorporate spatial hearing tests and speech-in-noise measures to provide a more comprehensive evaluation of functional auditory benefit. A further methodological limitation is that functional hearing outcomes were evaluated primarily using sound-field thresholds and the SSQ questionnaire. Although these measures provide useful information on auditory performance and patient-reported outcomes, they do not fully simulate complex real-world listening environments. Speech-in-noise tests such as the Matrix Sentence Test provide a more ecologically valid assessment of hearing performance and are increasingly recommended in hearing-device outcome studies. For example, Alberti et al. [21] demonstrated the utility of standardized speech-in-noise protocols for evaluating functional performance in hearing-aid users. Future prospective studies of VSB implantation should incorporate speech-in-noise assessments such as the Matrix Sentence Test to provide a more comprehensive evaluation of auditory benefit. Future prospective controlled studies with longer follow-up, bilateral VSB data, and objective spatial hearing assessment are warranted.

## 5. Conclusions

VSB implantation with SP provides effective auditory rehabilitation for older adults with sloping sensorineural hearing loss. The procedure yields meaningful high-frequency hearing gain, improved hearing-related quality of life, and significant tinnitus reduction while maintaining a favorable surgical safety profile. Restoration of auditory input through active middle ear implantation may also contribute to improved central auditory processing in older adults.

## Figures and Tables

**Table 1:** Patient demographics and surgical characteristics (*n* = 45).

Characteristic	Value
Age (years), mean ± SD	76.12 ± 5.3 (range 65–85)
Sex	Male, 45 (100%)
Implanted ear, *n* (%)	
Right	26 (57.8%)
Left	19 (42.2%)
Indication, *n* (%)	
Inadequate HA benefit	38 (84.4%)
HA intolerance	7 (15.6%)
Anticoagulant use, *n* (%)	8 (17.8%)
Warfarin	5 (11.1%)
DOAC	3 (6.7%)
Mean operative time (min), mean ± SD	40.2 ± 8.7
Bone reduction near incus required, *n* (%)	31 (68.9%)
Follow-up duration (months), mean ± SD	10.8 ± 3.6 (range 7–12)

DOAC, direct oral anticoagulant; HA, hearing aid; SD, standard deviation.

**Table 2:** Preoperative unaided and postoperative aided sound-field thresholds and functional gain (*n* = 45).

Frequency (Hz)	Unaided Threshold (dB HL)	Aided Threshold (dB HL)	Functional Gain (dB)
250	27.3 ± 5.1	27.1 ± 4.9	0.2 ± 2.1 (ns)
500	30.1 ± 6.2	30.0 ± 5.9	0.1 ± 2.3 (ns)
1000	43.6 ± 7.4	38.6 ± 6.8	5.0 ± 3.8 *
2000	58.2 ± 8.1	38.2 ± 7.3	20.0 ± 5.4 ***
4000	71.4 ± 9.3	41.2 ± 7.8	30.2 ± 6.1 ***
8000	80.3 ± 10.2	50.1 ± 9.4	30.2 ± 7.3 ***
4-PTA (0.5–4 kHz)	57.4 ± 8.3	35.6 ± 6.9	21.8 ± 5.2 ***

ns, not significant; * *p* < 0.05; *** *p* < 0.001 versus unaided condition (paired *t*-test). Data are mean ± SD. PTA, pure tone average.

## Data Availability

The original contributions presented in this study are included in the article. Further inquiries can be directed to the corresponding author.
